# A Randomized Clinical Trial Comparing Itraconazole and a Combination Therapy with Itraconazole and Potassium Iodide for the Treatment of Feline Sporotrichosis

**DOI:** 10.3390/jof10020101

**Published:** 2024-01-26

**Authors:** Erica Guerino dos Reis, Sandro Antonio Pereira, Luisa Helena Monteiro de Miranda, Raquel de Vasconcellos Carvalhaes de Oliveira, Marcel de Souza Borges Quintana, Paula Gonçalves Viana, Anna Barreto Fernandes Figueiredo, Cindy Caroline dos Santos Honorato, Gabriela Reis Pereira-Oliveira, Jéssica Nunes Silva, Tânia Maria Pacheco Schubach, Isabella Dib Ferreira Gremião

**Affiliations:** 1 Laboratory of Clinical Research on Dermatozoonoses in Domestic Animals, Evandro Chagas National Institute of Infectious Diseases, Oswaldo Cruz Foundation, Rio de Janeiro 21040-360, Brazil; erica.reis@bio.fiocruz.br (E.G.d.R.); sandro.pereira@ini.fiocruz.br (S.A.P.); paulaviana_rj@yahoo.com.br (P.G.V.); anna.figueiredo@ini.fiocruz.br (A.B.F.F.); cindy.honorato@ini.fiocruz.br (C.C.d.S.H.); gabi_reis2004@yahoo.com.br (G.R.P.-O.); jessicanunes7@gmail.com (J.N.S.); tpschu@yahoo.com.br (T.M.P.S.); 2 Sydney School of Veterinary Science, The University of Sydney, Sydney, NSW 2006, Australia; luisa.miranda@sydney.edu.au; 3 Laboratory of Clinical Epidemiology, Evandro Chagas National Institute of Infectious Diseases, Oswaldo Cruz Foundation, Rio de Janeiro 21040-361, Brazil; raquel.vasconcellos@ini.fiocruz.br (R.d.V.C.d.O.); marcel.quintana@ini.fiocruz.br (M.d.S.B.Q.)

**Keywords:** sporotrichosis, *Sporothrix*, cats, itraconazole, potassium iodide, treatment, combination therapy

## Abstract

Feline sporotrichosis is an endemic disease with high occurrence in Brazil. Itraconazole (ITZ) remains the drug of choice for treating this disease in cats, despite the increasing reports of therapeutic failure. A controlled, randomized clinical trial was performed on 166 naive cats with sporotrichosis to assess the effectiveness and safety of the combination therapy with ITZ and potassium iodide (KI) compared with ITZ monotherapy. Cats were randomly allocated into two treatment groups: G1—ITZ 100 mg/cat/day—and G2—ITZ 100 mg/cat/day + KI 2.5–20 mg/kg/day. Cats treated in G2 presented 77% more risk of reaching a clinical cure (a positive effect) than those treated in G1, even when controlled by negative predictors. The survival curves of the two treatment protocols indicate that a clinical cure was achieved faster in G2. An increase in the KI dose was necessary in 28 cats due to the persistence of clinical signs. Adverse reactions were equally frequent in both groups and manageable with a temporary drug suspension and/or a hepatoprotective therapy. The combination therapy was associated with a higher cure rate and a shorter treatment time, suggesting that ITZ+KI arises as a better option for treating feline sporotrichosis and should be considered the first-line treatment, especially in the presence of negative predictors.

## 1. Introduction

*Sporothrix brasiliensis* and *Sporothrix schenckii* are the main causative agents of feline sporotrichosis [1,2,3,4,5,6], with cases mostly reported in South America, Asia, and the United States [1,7,8,9,10]. *S. brasiliensis* is the main etiological agent of sporotrichosis in Brazil, where epizootic as well as zoonotic transmission related to cats have been reported over the last twenty-five years [2,11,12,13,14,15,16], although the isolation of this species from the environment suggests that sapronotic transmission can also occur [17]. 

*S. brasiliensis* is the most virulent species [11] and related to atypical and severe clinical manifestations in cats and humans, with increasing virulence over time [18,19,20]. Recently, new cases involving *S. brasiliensis* have been reported in Argentina, Chile, Paraguay, the United States of America, and the United Kingdom [21,22,23,24,25], indicating a propagation of this species. 

Cats are the most susceptible animal species to *Sporothrix* infection, often developing severe forms of the disease [2,26,27]. Clinical manifestations range from a single to multiple skin lesions, which can progress to disseminated systemic forms [28]. Nodules and ulcers are the most common lesion types, followed by respiratory signs (dyspnea, sneezing, nasal discharge) and mucosal involvement (nasal, ocular) [29,30]. The presence of lesions on the nasal bridge, nasal mucosa, respiratory signs, and lesions in multiple sites are considered negative predictors that may hinder a complete cure [29,31,32].

The early diagnosis of the disease combined with a fast initiation of treatment represents an important disease control measure, as it may induce a quick reduction in the fungal burden [33]. On the other hand, the increased virulence of *S. brasiliensis*, unrestricted access of cats to outdoors, and an inappropriate treatment protocol could lead to longer treatment times, therapeutic failure, or even abandonment, which seems to be an issue for the epidemiological control [30].

Many potential drug candidates showed in vitro activity against *Sporothrix* [15,34,35,36,37,38], but in spite of that, there are few available antifungal agents that are effective in vivo against feline sporotrichosis [30].

The therapeutic options for feline sporotrichosis are most often used as monotherapy regimens, and itraconazole (ITZ) remains the drug of choice for treating the disease [30]. Due to the increasing number of cases that are refractory to ITZ [36,39,40], the combination of antifungal agents should be considered to achieve synergy [30]. In addition, alternative therapies such as intralesional amphotericin B, cryosurgery, intranasal clotrimazole spray, local laser therapy, and photodynamic therapy have been associated with an oral antifungal agent [41,42,43,44,45], but there are some limitations due to its clinical indication. 

The combination therapy with KI has been described as the best therapeutic option for treating feline sporotrichosis; however, this is based on case series studies [40,46]. Randomized clinical trials (RCTs) provide the highest level of evidence for evaluating the effectiveness of a treatment, since they are designed to reduce bias and systematic errors [47,48], which is essential for evidence-based veterinary medicine (EBVM) [49]. 

Thus, the aim of this study was to assess whether ITZ+KI is more effective and safer than the conventional treatment (ITZ) through a controlled randomized clinical trial.

## 2. Materials and Methods

This was a prospective, non-blinded, randomized, controlled clinical trial comparing monotherapy with ITZ and the combination therapy with ITZ+KI in owned cats with sporotrichosis. The study was conducted at the Laboratory of Clinical Research on Dermatozoonoses in Domestic Animals (Lapclin-Dermzoo), INI, Fiocruz, and the cats were enrolled from 2013 to 2016. 

The protocol and the informed consent were reviewed and approved by the Ethics Commission on the Use of Animals (CEUA/Fiocruz), license number LW37/2012.

### 2.1. Inclusion, Exclusion, and Elimination Criteria

Cats seen at Lapclin-Dermzoo/INI/Fiocruz and suspected of having sporotrichosis were assessed by two veterinarians at day 0 to determine eligibility for the study. Inclusion criteria comprised cats of any breed, older than 6 months, weight > 3 kg, and a clinical and cytopathological diagnostic of sporotrichosis. The exclusion criteria were previous systemic antifungal therapy, owners that did not sign informed consent, and concurrent pregnancy at day 0. 

Cats that fulfilled eligibility criteria were randomized into a treatment group, but they were subsequently removed from the study if *Sporothrix* sp. could not be isolated in culture from cutaneous lesion or nasal swabs, or if the owners did not return for the second visit (declined treatment). These data were not computed and analyzed in the trial.

### 2.2. Study Procedure

Cats enrolled in the study underwent clinical examination, collection of clinical specimens, and photographic data. Anamnesis was carried out in every follow-up visit.

The clinical examination consisted of the following procedures: evaluation of health condition (good, fair, and poor), inspection of the skin and mucous membranes (conjunctive, nasal, oral, genital, and anal), palpation of lymph nodes (mandibular, parotid, axillary, and popliteal), and body weigh assessment. The veterinarians also inspected the nasal cavity and checked for the presence of respiratory signs. The time elapsed between the onset of clinical signs and the first appointment was based on the information provided by the owners. 

To estimate the level of dissemination of cutaneous lesions, the cats were divided into three groups: L1 (skin lesions at one site), L2 (skin lesions at two non-adjacent sites), and L3 (skin lesions at three or more non-adjacent sites) [29]. 

Exudate from ulcerated lesions or secretion from nasal cavities were collected using sterile swabs and seeded on to Sabouraud dextrose agar and Mycobiotic agar (Difco™; Becton, Dickinson and Company, Sparks, Sparks Glencoe, MD, USA), incubated at 25 °C, and observed for four weeks for fungal growth. Suspected isolates were subcultivated on potato dextrose agar medium (Difco™) at 25 °C for macroscopic and microscopic morphological studies. Dimorphism was demonstrated by conversion to the yeast-like form on brain heart infusion agar medium (Difco™) at 37 °C. T3B PCR fingerprinting was used for molecular identification of the *Sporothrix* species [50].

Blood samples were collected for a complete blood count and biochemistry analysis (urea, creatinine, alanine aminotransferase [ALT], aspartate aminotransferase [AST]). In addition, serum samples from all cats were tested for the presence of antibodies against feline immunodeficiency virus (FIV) and antigen of feline leukemia virus (FeLV) by Snap FIV/FeLV Combo Test™ (IDEXX Laboratories Inc., Westbrook, ME, USA), according to manufacturer’s instructions.

### 2.3. Sample Size and Randomization 

An estimated sample of 74 cats in each group was established to detect a difference of 25% in clinical cure between treatments, considering a dropout rate of 20%, an alpha (α) of 0.05, and a power of 0.8.

Each cat was assigned a number based on admission order. According to this number, they were randomly allocated (1:1 ratio) to group G1 (ITZ) or G2 (ITZ+KI), using the random function of the software Statistical Package for the Social Sciences v16.0. 

Cat owners and the veterinary staff were non-blinded to the allocated treatment.

### 2.4. Treatment

Cats randomized to G1 received only ITZ 100 mg (Prati-Donaduzzi, Toledo (PR), Brazil) in a blister pack. Owners were instructed to administer one capsule per day, directly into the oral cavity or mixed with canned cat food. 

Cats randomized to G2 received ITZ 100 mg (Prati-Donaduzzi) plus compounded KI (about 2.5 mg/kg) in a different capsule pack. Owners were advised to administer one capsule of ITZ 100 mg per day and the KI every other day for the first 7 days. After that, KI should be administered per day. They were also instructed to provide the drugs at the same time, directly into the oral cavity or mixed with canned cat food. The proposed dose for KI in cats should never be managed all at once, but gradually increased as previously reported [46,51].

Drugs were dispensed according to each treatment and in the required amount to last until the next appointment. Owners were guided to call the veterinary staff in case of clinical adverse reaction (CAR) and hepatotoxicity, such as loss of appetite, lethargy, vomiting, diarrhea, or jaundice or if they had any doubt about the treatment. Furthermore, they were requested to return with patients to the scheduled follow-up visit within 30 days until treatment outcome.

All procedures, laboratorial tests, and drugs were supplied free-of-charge. Owners had to provide informed consent prior to enrolment and were free to withdraw at any point without any loss to the cats’ treatment and care. 

### 2.5. Follow-Up Procedures

The follow-up appointments were carried out monthly for anamnesis, clinical examination, laboratorial tests (complete blood count and biochemistry analysis), and photographic documentation. During these visits, questions about adverse reactions were asked. Clinical signs were recorded and compared with the previous appointment. 

Cats presenting hyporexia or anorexia combined with body loss of >10% or the association of CAR and elevated serum aminotransferase (ESA) had a temporary suspension of the drug(s). Drug suspension was for a minimum of seven days for G1. In G2, both drugs were suspended at the same time, but the reintroduction of ITZ occurred on the eighth day and the KI on the eleventh, in an attempt to detect which drug was causing the unwanted effect. The maximum period for the temporary interruption of the drug(s) was set as 30 days. A hepatoprotective therapy with oral silymarin 30 mg/kg/day was prescribed for persistence of CAR and ESA. 

In cases of persistence of the initial skin/mucosal lesion(s), persistence of respiratory signs, or worsening of the lesion(s), cats from G2 had the dose of KI increased by increments of about 2.5 mg/kg at the follow-up visit up to a satisfactory clinical response or until the dose reached 20 mg/kg maximum. 

Clinical cure was defined as a complete healing of the skin/mucosal lesions and/or remission of respiratory signs. After clinical cure, the therapy was maintained for about a month, which corresponded to the discharge assessment. Cats that did not present any improvement in two consecutive follow-up visits were considered treatment failure, as were cats that were incapable of tolerating KI 2.5 mg/kg and/or ITZ 100 mg. The inability to tolerate the drug(s) was defined by the persistence of CAR and ESA after the temporary suspension of the drug(s) and reintroduction of ITZ or ITZ+KI. 

### 2.6. Statistical Analysis

For exploratory analysis of the data, we calculated the frequency distribution for categorical variables and summary measures (median—MD, interquartile range—IQR) for quantitative variables.

The Cox semiparametric regression model was used to identify factors associated with clinical cure. Time was measured in weeks from inclusion to the event (clinical cure or censoring). The cure was the event of interest, while censorship occurred due to other outcomes (therapeutic failure, loss of follow-up, and death). The treatment groups were monotherapy (G1) and combination therapy (G2). In the multivariate regression, significant predictors from univariate regression were chosen as adjustment variables. Non-significant variables were sequentially removed from the model. Hazard Ratios (HRs) with 95% confidence intervals were provided for interpretation. An HR = 1 suggests no difference in the cure rate, while HR > 1 implies increased cure rate (positive effect), and HR < 1 indicates a smaller cure rate (negative effect). The Cox proportionality assumption was not rejected by the Schoenfeld test. 

The median time until clinical cure in each treatment group was estimated using the Kaplan–Meier survival curves and the Hazard Ratio (HR) and its respective confidence interval (CI), calculated from the Cox model. The survival curve of Kaplan–Meier serves as a metric for assessing the probability of progression of non-cure over time.

The log-rank test was used to compare the distribution of time up to the occurrence of an adverse reaction to drugs (CAR or ESA). 

A *p*-value < 0.05 was considered statistically significant. 

All analyses were performed using the R v4.2.1 software (R Foundation for Statistical Computing, Vienna, Austria). 

## 3. Results

**Figure 1:**
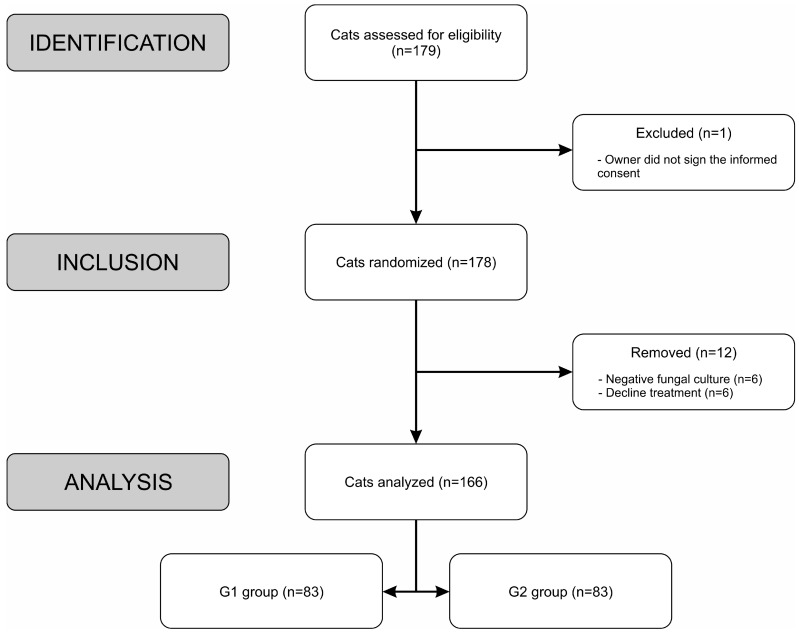
Inclusion flow chart illustrating the recruitment process of the cats, Rio de Janeiro, Brazil.

One hundred and seventy-nine cats were evaluated. The recruitment process of the eligible cats is illustrated in Figure 1.

### 3.1. Exploratory Analysis

**Figure 2:**
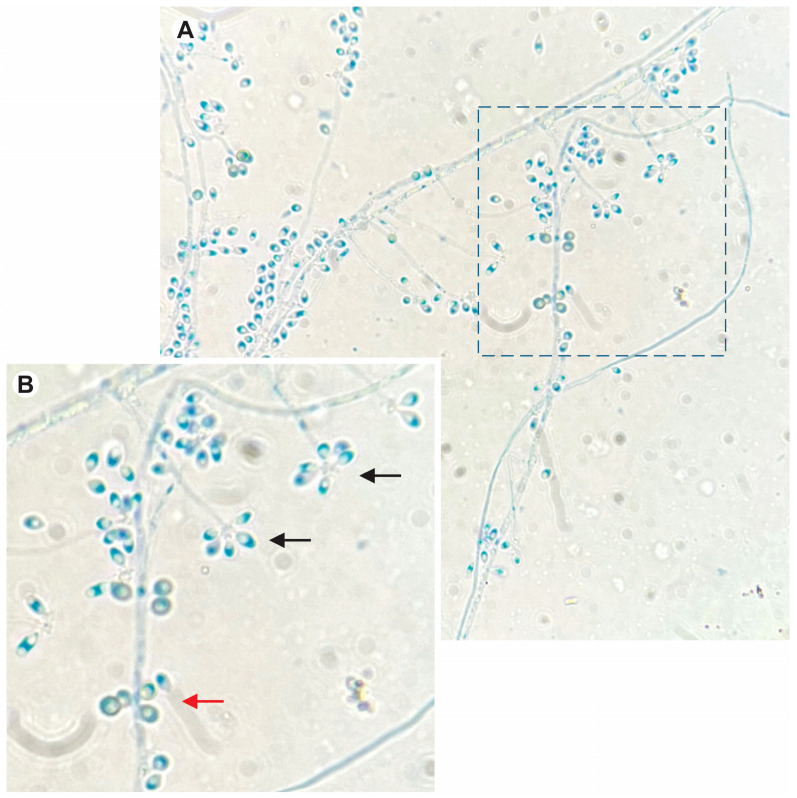
(**A**) Micromorphology of the filamentous form of *S. brasiliensis*, stained with cotton blue lactophenol, showing septate hyaline hyphae with sympodial conidiophores (magnification 1000×). (**B**) Close-up picture of the two types of conidia observed: hyaline (black arrow) and dematiaceous conidia (red arrow).

All cats were residents in Rio de Janeiro state. Most of them were male (73.4%), mixed breed (91.5%), had access to the outdoors (78.3%), and were in good overall condition (88.5%). Molecular identification was performed in 21 isolates of clinical samples, and all of them were identified as *S. brasiliensis.* Figure 2 shows the micromorphology of the filamentous form of *S. brasiliensis.*

The clinical characteristics of the cats are shown in Table 1. The groups were equivalent in terms of age and weight, and they had the same median interval between the onset of clinical signs (information provided by the owner) and the first appointment. Skin lesions were detected in all cases, respiratory signs in 31.9%, and mucosal lesions in 29.5%. 

The overall median dose of ITZ in the trial was 24.4 mg/kg (IQR = 21.2–27.7 mg/kg), while the dose of KI was 2.6 mg/kg (IQR = 2.3–2.9 mg/kg). 

Twenty-eight cats in G2 had their dose of KI increased (Table 2), and two cats required a dose reduction due to persistent clinical adverse effects at the proposed dose. The maximum dose of KI reached in this trial was 12.5 mg/kg. A borderline effect was noted when evaluating the influence of a 10-unit increment in the KI dosage on the cure rate (HR (95%CI) = 0.14 (0.02–1.11), *p*-value = 0.06).

**Figure 3:**
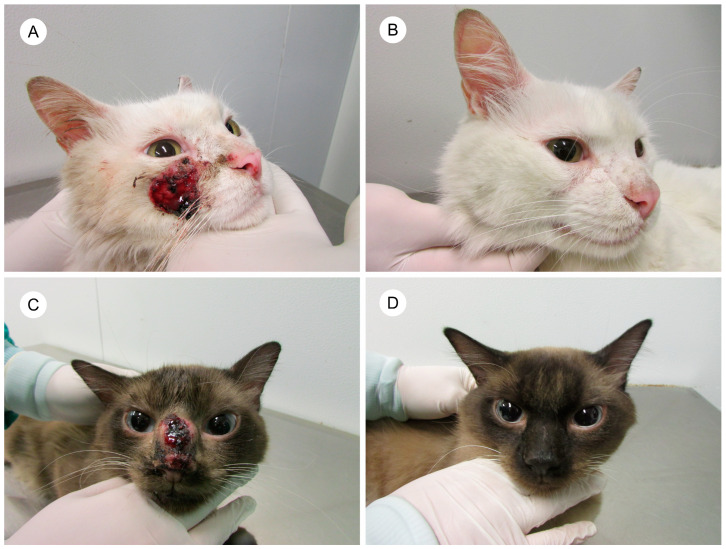
(**A**) Cat with sporotrichosis with an extensive ulcer with hematic crust on the right lateral face and erosions on the nose and in the nasal philtrum before treatment. (**B**) The skin lesions have resolved after ITZ monotherapy. (**C**) Cat with sporotrichosis with an ulcerated nodule with presence of hematic crust on the nasal bridge and nasal planum. (**D**) The lesion has resolved, and the nasal bridge returned to normal after ITZ+KI treatment.

**Figure 4:**
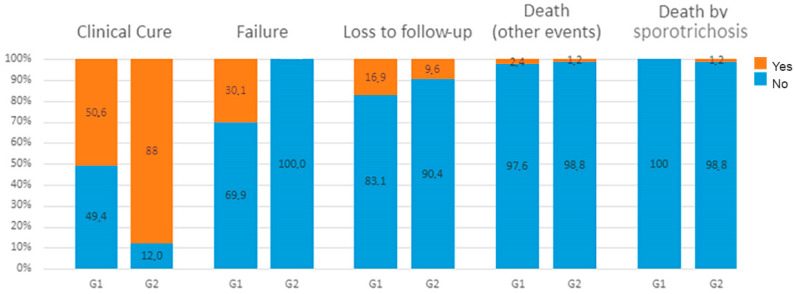
Frequency of outcome of the 166 cats with sporotrichosis, according to treatment group, Rio de Janeiro, Brazil.

One hundred and fifteen cats achieved clinical cure (67.2%) and were discharged from the therapeutic protocol (Figure 3). The cure rate was 50.6% in G1 and 88% in G2. Figure 4 shows the frequency of outcome of the 166 cats who were included in the trial.

In total, 25 G1 cats were censored due to therapeutic failure, which was assigned to disease stagnation (*n* = 16), a worsening of clinical signs (*n* = 7), and a severe adverse drug reaction (*n* = 2). 

The FIV and/or FeLV co-infection seemingly did not affect the treatment response in this sample. Of the 31 co-infected cats, 23 were clinically cured (G1: *n* = 8/13; G2: *n* = 15/18), 5 were lost to follow-up (G1: *n* = 2/13; G2: *n* = 3/18), and 3 failed treatment (G1: *n* = 3/13; 10%).

**Figure 5:**
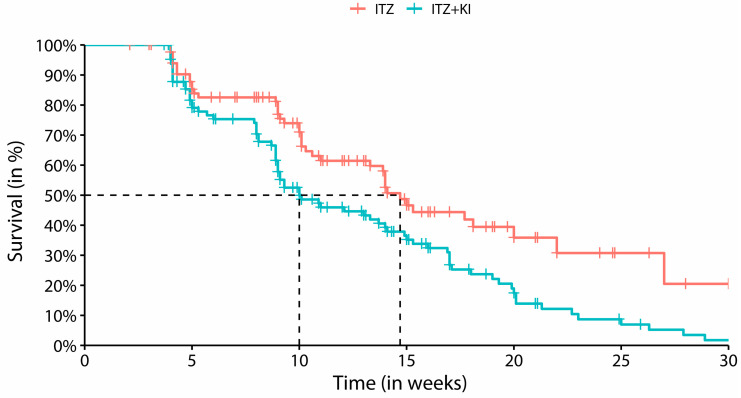
Kaplan–Meier survival curves comparing treatment time between cats treated with ITZ (G1) and ITZ+KI (G2). The median time until cure was 14.9 weeks for ITZ (G1) and 10 weeks for ITZ+KI (G2). The tick marks indicate censured data.

The comparison between the survival curves (Figure 5) for the time to clinical cure of the two protocols indicates that a clinical cure was achieved faster in cats treated with the combination therapy (G2) than in those who were treated with ITZ monotherapy (G1) (HR (95%CI) = 1.768 (1.207, 2.588), *p*-value < 0.01). After cure, the therapy was maintained for about a month.

### 3.2. Cox Regression Model

The following clinical–epidemiological variables were entered into the univariate Cox model to investigate the independent risk factors for clinical cure: treatment group, general condition, sex, age, respiratory signs, temporary drug suspension, lymphangitis, lymphadenomegaly, mucosal lesion, access to the outdoors, distribution of skin lesions, neutering, and FIV/FeLV infection.

The exposure variable—treatment group—showed significance in univariate and multivariate Cox models (Table 3). 

Cats in G2 (ITZ+KI) presented a 77% higher risk of reaching the event of clinical cure (a positive effect) than cats in G1 (ITZ), even when controlled by other factors, including negative predictors such as the presence of “respiratory signs” and “mucosal lesions”. In addition, the Cox model shows that the absence of lymphangitis is a negative predictor in time to achieving clinical cure.

### 3.3. Safety

The frequency of an adverse drug reaction (ADR) was similar in both groups regarding CAR and ESA. 

An adverse clinical reaction was noted in 35 cats (42.2%) from G1 and in 39 cats (47.0%) from G2, with hyporexia being the most frequent one (G1: *n* = 23/35; G2: *n* = 27/39). Serum transaminases were elevated in 50 cats from G1 (60.2%) and in 43 cats (51.8%) from G2 (Table 4).

In this study, the distribution of the time up to the occurrence of CAR (*p* = 0.493) or ESA (*p* = 0.096) did not differ between groups. 

In total, 37 cats had their therapy temporarily suspended (G1: *n* = 15, 18.0%; G2: *n* = 22, 26.5%) due to clinical signs of hepatotoxicity (G1: *n* = 4; G2: *n* = 9) or these signs combined with an elevation of serum transaminases (G1: *n* = 12; G2: *n* = 12). Signs of CAR were reversible within 7 to 20 days (MD = 7 days) of temporary drug suspension, and the transaminases alterations returned to pre-treatment levels after drug suspension and/or oral hepatoprotective therapy.

Two cats from G1 were censured due to severe CAR (anorexia, weight loss, apathy, jaundice) and marked elevation of ALT. They were indexed as treatment failure due to an inability to tolerate the drug.

## 4. Discussion

In the hyperendemic scenario of Rio de Janeiro, the lack of public health polices to curb sporotrichosis combined with the absence of free-of-charge antifungal medications, the cat’s high zoonotic potential to transmit *S. brasiliensis*, and the challenging nature of treating cats orally and keeping them indoors may be some of the reasons for having an increased number of human and feline sporotrichosis cases [30,33]. Furthermore, educational measures to ensure responsible ownership, mass neutering, early diagnosis, and mass treatment can slow the spread of the disease [20,30,54]. 

The combination of ITZ and KI has been considered an important option for the treatment of refractory cases [40], as well as for naïve cats [46], especially for those presenting with multiple cutaneous lesions, nasal mucosa lesions, and/or respiratory signs [30,46]. To evaluate the efficacy and safety of this treatment protocol and consider it as first-line treatment for feline sporotrichosis, we carried out a clinical trial comparing it with the conventional protocol (ITZ monotherapy).

In this trial, the combined therapy with ITZ+KI increased the rate of clinical cure up to 77 times. This finding is consistent with previous studies that noted an increased likelihood of recovery from sporotrichosis with the use of the combination therapy for both naïve cats [46] and cats that did not respond to ITZ monotherapy [40]. Respiratory signs, mucosal lesions, skin lesions on three or more sites (L3), and not being neutered are, in fact, obstacles to achieving a clinical cure, as previously reported [29,31,32]. Interestingly, lymphangitis seems to be a positive predictor in cats, which has not been reported so far, differing from human sporotrichosis cases [55,56]. In addition, ITZ+KI therapy has also been shown to be effective in the healing and in the prompt control of the fungal burden in skin lesions from cats with sporotrichosis [33]. In human sporotrichosis, ITZ combined with KI is used in cases with severe or ITZ-refractory sporotrichosis [57], as well as for the treatment of other fungal infections [58,59]. 

Although this trial was controlled and randomized, we did not blind the groups, as the prescribed KI dose must be escalated to avoid toxicity [46,51]. An increase in the KI dose was expected in case of a non-response to the initial dose. The overall KI dose in the present study was lower than that reported in a study conducted with refractory cats [40], which is somehow expected, considering the shorter time that has elapsed between the onset of clinical signs and the start of treatment in naïve cats (m = 8 weeks). The higher rate of nasal lesions and respiratory signs in refractory cats could have contributed to the higher KI dose, since nasal lesions, respiratory signs, and lesions in multiples sites (group L3) are predictors of severity in feline sporotrichosis and are linked to an increased risk of negative outcomes. Although Retrovirus/*Sporothrix* co-infection can lead to important changes in the immunological balance of cats [60], in this study, the co-infection did not represent an obstacle to clinical cure, which is consistent with other findings [31,32,46,60]. 

Both therapeutic regimens were reasonably tolerated by the cats in this study. The CAR of ITZ monotherapy (42.2%) was higher than those observed in a retrospective study of feline sporotrichosis using this azolic (30.9%) [32]. However, it is important to emphasize that the dose range of ITZ in this trial was higher (21.2–27.7 mg/kg) than the one previously reported (8.3–27.7 mg/kg) [32]. CAR and increased ALT are considered a dose-dependent effect to ITZ administration and have also been reported in cats with sporotrichosis under KI therapy [40,46,51]. 

Regarding KI, the tolerance threshold for the drug in cats may range from 2.5 to 20 mg/kg [51], even though in this study, the maximum dose reached was 12.5 mg/kg. The frequency of CAR in this study (47%) was lower than the one reported for refractory cats (76.3%) [40], which could be explained by the higher dose of KI and the previous treatment with ITZ (MD = 16 weeks) in those cats compared to the present study. In this trial, the ADRs were successfully managed with a temporary drug suspension and/or oral hepatoprotective therapy as previously reported [46]. There was no statistical difference between CAR and ESA in both treatment groups, demonstrating that the combined therapy is as safe as ITZ monotherapy. 

The criterion for a cure of feline sporotrichosis is clinical and determined by the complete remission of all clinical signs [30]. It is important to continue the treatment for at least 1 month after that to minimize the risk of recurrence [30,46]. Although the median time up to a clinical cure differed between G1 and G2, it was consistent with the medians that have already been reported in studies involving treatment with ITZ [31,61] and ITZ+KI [46]. In cats treated with ITZ monotherapy, a shorter treatment might be tied to the early intervention with the use of an approved generic capsule of the drug. Compounded formulations of ITZ, on the other hand, are absorbed poorly and inconsistently [62], so we do not recommend their use in cats. While branded ITZ may be cost-prohibitive in low socioeconomic endemic areas such as in Brazil, the generic drug is a satisfactory alternative [30], as demonstrated in this trial.

Although these results endorse the previous reports on the beneficial use of KI for the treatment of feline sporotrichosis, the mechanism of action of KI is still to be determined. While a direct antimicrobial effect seems unlikely, immune-related effects are more well-accepted but still controversial [63,64]. A shortened time to remission of all clinical signs in cats treated with the combined therapy may be related to the anti-inflammatory effect of KI linked to cytokine regulation [65], and/or to a beneficial interaction between ITZ and KI that has not been fully elucidated yet. In addition, excessive iodine supplementation in humans, as well as in cats, may affect the thyroid function, which might have an impact on the host immune response, but this has not been explored with the use of KI in the treatment of sporotrichosis [66,67,68,69,70,71].

Cats receiving the combined therapy had shown increased cure rates, a lower rate of drop-out, and a fast onset of action, suggesting that this treatment regimen (ITZ+KI) should be considered the first-line treatment, notably in the presence of negative predictors. Further studies are still encouraged to investigate the mechanisms behind the successful use of KI in the treatment of feline sporotrichosis. We believe that this can provide valuable information on the use of KI and boost the confidence of veterinarians at prescribing this drug for cats.

## Figures and Tables

**Table 1:** Clinical characteristics of the 166 cats with sporotrichosis, according to treatment group, Rio de Janeiro, Brazil.

Variables	G1-ITZ (*N* = 83)	G2-ITZ+KI (*N* = 83)
Sex, *n* (%)		
Male	58 (69.9%)	64 (77.1%)
Female	25 (30.1%)	19 (22.9%)
Clinical form, *n* (%)		
Cutaneous	51 (61.4%)	56 (67.5%)
Cutaneous/Mucosal	32 (38.6%)	27 (32.5%)
Distribution of skin lesions, *n* (%)		
L1	27 (32.5%)	26 (31.3%)
L2	22 (26.5%)	22 (26.5%)
L3	34 (41.0%)	35 (42.2%)
Respiratory signs, *n* (%)	30 (36.1%)	23 (27.7%)
Lymphadenomegaly, *n* (%)	69 (83.1%)	67 (80.7%)
Lymphangitis, *n* (%)	16 (19.3%)	13 (15.7%)
Access to outdoors, *n* (%)	68 (81.9%)	62 (74.1%)
Neutering, *n* (%)	36 (43.4%)	45 (54.2%)
FIV Ab and FeLV Ag *, *n* (%)	13 (8.07%)	18 (11.18%)
Age in months, MD (IQR) **	24 (24–48)	24 (15–36)
Weight in Kg, MD (IQR) **	4 (3.5–4.7)	4 (3.6–4.5)
Time interval between the onset of clinical signs in weeks, MD (IQR) **	8 (4–12)	8 (4–12)

* FIV = feline immunodeficiency virus antibody; FeLV= feline leukemia virus antigen. ** MD = median, IQR = interquartile range.

**Table 2:** Potassium iodide dose adjustment in 28 cats from G2 group, Rio de Janeiro, Brazil.

Variable		Cats with Increasing Dose		KI mg/kg
		*n*	%	Range
Persistent Lesion	Skin	15	53.6	2.5–9.5
	Mucosal	2	7.1	3.1–8.1
	Skin and mucosal	11	39.3	2.5–12.5

**Table 3:** Univariate and multivariate Cox model analyses of 166 cats with sporotrichosis, Rio de Janeiro, Brazil.

Predictors	Category	Clinical Cure		Crude HR (95% CI)	Adjusted HR (95% CI)
		Yes, *n* (%)	No, *n* (%)		
Treatment group	ITZ	42 (50.6)	41 (49.4)	1	1
	ITZ+KI	73 (88)	10 (12)	1.77 (1.21–2.59)	1.77 (1.2–2.62)
Respiratory signs	Yes	31 (58.5)	22 (41.5)	1	1
	No	84 (74.3)	29 (25.7)	3 (1.74–5.17)	2.09 (1.14–3.83)
Mucosal lesions	Yes	32 (54.2)	27 (45.8)	1	1
	No	83 (77.6)	24 (22.4)	2.36 (1.56–3.58)	1.74 (1.09–2.78)
Distribution of skin lesions	L3	41 (59.4)	28 (40.6)	1	1
	L2	35 (79.5)	9 (20.5)	1.76 (1.12–2.78)	2.08 (1.3–3.32)
	L1	39 (73.6)	14 (26.4)	1.79 (1.15–2.81)	2.24 (1.39–3.61)
Lymphangitis	Yes	23 (79.3)	6 (20.7)	1	1
	No	92 (67.2)	45 (32.8)	0.55 (0.34–0.87)	0.46 (0.28–0.76)
Neutering	No	48 (58.5)	34 (41.5)	1	1
	Yes	67 (82.7)	14 (17.3)	1.78 (1.22–2.58)	1.61 (1.09–2.37)

Abbreviations: HR = hazard Ratio, CI = confidence interval.

**Table 4:** Evaluation of serum aminotransferase in 166 cats treated for sporotrichosis, according to treatment group, Rio de Janeiro, Brazil.

Aminotransferase Levels	G1-ITZ (*N* = 83)	G2-ITZ+KI (*N* = 83)	Unit	Reference Value
Normal	33	40	U/L	AST (6–83) [52] ALT (26–43) [52]
Mild elevation (ALT and/or AST)	43	39	U/L	<5 times the upper reference range [53]
Moderate elevation (ALT)	5	4	U/L	5–10 times the upper reference range [53]
Marked elevation (ALT)	2	0	U/L	>10 times the upper reference range [53]

Abbreviations: ALT: Alanine aminotransferase; AST: Aspartate aminotransferase.

## Data Availability

Data are contained within the article.
